# Increasing the Efficacy of Seproxetine as an Antidepressant Using Charge–Transfer Complexes

**DOI:** 10.3390/molecules27103290

**Published:** 2022-05-20

**Authors:** Walaa F. Alsanie, Abdulhakeem S. Alamri, Hussain Alyami, Majid Alhomrani, Sonam Shakya, Hamza Habeeballah, Heba A. Alkhatabi, Raed I. Felimban, Ahmed S. Alzahrani, Abdulhameed Abdullah Alhabeeb, Bassem M. Raafat, Moamen S. Refat, Ahmed Gaber

**Affiliations:** 1 Department of Clinical Laboratories Sciences, The Faculty of Applied Medical Sciences, Taif University, Taif 21944, Saudi Arabia; w.alsanie@tu.edu.sa (W.F.A.); a.alamri@tu.edu.sa (A.S.A.); m.alhomrani@tu.edu.sa (M.A.); 2 Centre of Biomedical Sciences Research (CBSR), Deanship of Scientific Research, Taif University, Taif 21944, Saudi Arabia; a.s.zahrani@tu.edu.sa; 3 College of Medicine, Taif University, Taif 21944, Saudi Arabia; hmyami@tu.edu.sa; 4 Department of Chemistry, Faculty of Science, Aligarh Muslim University, Aligarh 202002, India; sonamshakya08@gmail.com; 5 Department of Medical Laboratory Technology, Faculty of Applied Medical Sciences in Rabigh, King Abdulaziz University, Jeddah 21589, Saudi Arabia; hhabeeballah@kau.edu.sa; 6 Department of Medical Laboratory Sciences, Faculty of Applied Medical Sciences, King Abdulaziz University, Jeddah 21589, Saudi Arabia; halkhattabi@kau.edu.sa (H.A.A.); faraed@kau.edu.sa (R.I.F.); 7 Center of Excellence in Genomic Medicine Research (CEGMR), King Abdulaziz University, Jeddah 21589, Saudi Arabia; 8 King Fahd Medical Research Centre, Hematology Research Unit, King Abdulaziz University, Jeddah 21589, Saudi Arabia; 9 Center of Innovation in Personalized Medicine (CIPM), 3D Bioprinting Unit, King Abdulaziz University, Jeddah 21589, Saudi Arabia; 10 National Centre for Mental Health Promotion, Riyadh 11525, Saudi Arabia; aalhabeeb@ncmh.org.sa; 11 Department of Radiological Sciences, College of Applied Medical Sciences, Taif University, Taif 21944, Saudi Arabia; bassemraafat@tu.edu.sa; 12 Department of Chemistry, College of Science, Taif University, Taif 21944, Saudi Arabia; 13 Department of Biology, College of Science, Taif University, Taif 21944, Saudi Arabia

**Keywords:** seproxetine, antidepressant, charge transfer, *π*-acceptors, DFT

## Abstract

The charge transfer interactions between the seproxetine (SRX) donor and *π*-electron acceptors [picric acid (PA), dinitrobenzene (DNB), p-nitrobenzoic acid (p-NBA), 2,6-dichloroquinone-4-chloroimide (DCQ), 2,6-dibromoquinone-4-chloroimide (DBQ), and 7,7′,8,8′-tetracyanoquinodi methane (TCNQ)] were studied in a liquid medium, and the solid form was isolated and characterized. The spectrophotometric analysis confirmed that the charge–transfer interactions between the electrons of the donor and acceptors were 1:1 (SRX: *π*-acceptor). To study the comparative interactions between SRX and the other *π*-electron acceptors, molecular docking calculations were performed between SRX and the charge transfer (CT) complexes against three receptors (serotonin, dopamine, and TrkB kinase receptor). According to molecular docking, the CT complex [(SRX)(TCNQ)] binds with all three receptors more efficiently than SRX alone, and [(SRX)(TCNQ)]-dopamine (CTcD) has the highest binding energy value. The results of AutoDock Vina revealed that the molecular dynamics simulation of the 100 ns run revealed that both the SRX-dopamine and CTcD complexes had a stable conformation; however, the CTcD complex was more stable. The optimized structure of the CT complexes was obtained using density functional theory (B-3LYP/6-311G++) and was compared.

## 1. Introduction

Depression is the most common mental illness, affecting roughly 322 million people worldwide [1]. Depression is the main cause of disability and the fourth major contributor to the global illness burden [2]. Antidepressants are the third most commonly sold class of therapeutic drugs worldwide [3]. The majority of these treatments are based on chemicals that target the serotonin (5-hydroxytryptamine (5-HT): a group of G protein-coupled receptor and ligand-gated ion channels found in the central and peripheral nervous systems) transporter, a single protein in the brain. Selected serotonin reuptake inhibitors (SSRIs), which block 5-HT reuptake, account for around 80% of all antidepressants on the market [3]. Other antidepressants, such as serotonin and noradrenaline reuptake inhibitors, as well as traditional tricyclic antidepressants (e.g., amitryptyline, clomipramine, imipramine), prevent noradrenaline reuptake. Indeed, compared to tricyclic medicines, the success of selective serotonin reuptake inhibitors is mostly due to their safety, tolerability, and lack of severe side effects, which enhances patient compliance and quality of life [3].

Although seproxetine (SRX, also known as S-norfluoxetine) is classified as a selective serotonin reuptake inhibitor, its inhibitory action extends beyond serotonin transporters to dopamine transporters (DAT) and 5-HT2A/2C receptors [4]. It is the active N-demethylate metabolite of the commonly prescribed antidepressant fluoxetine and is deemed more potent than the parental compound itself [5]. The 5-HT(2A) and 5-HT(2C) receptors belong to the G-protein-coupled receptor (GPCR) superfamily. GPCRs interact with G-proteins to transmit extracellular signals to the inside of cells. The 5-HT(2A) and 5-HT(2C) receptors are involved in the effects of a wide range of drugs on anxiety, sleep patterns, depression, hallucinations, schizophrenia, dysthymia, eating behavior, and neuro-endocrine processes [6].

As SRX was found to be a 20 times more potent serotonin inhibitor than its sister enantiomer R-norfluoxetine, significant research efforts were focused on this drug in the 1990s [7]. However, serious cardiac side effects, such as QT prolongation (a measure of delayed ventricular repolarisation), halted further development [4,8]. The potency of SRX as a serotonin inhibitor should not be ignored, and an effort must be taken to chemically modify (charge–transfer complexation) SRX for a better serotonin inhibitor while suppressing the drawback.

Charge–transfer (CT) complexation, or electron–donor transfer, is a crucial aspect of biochemical and biological processes such as drug design, enzyme catalysis, and ion sensing [9]. The pharmacodynamics and thermodynamics of therapeutic substances and biological processes in the human body are studied using charge–transfer complexation interactions [10,11,12,13,14]. In biological systems, charge–transfer complexes may play a crucial function. Extensive research has been carried out on charge–transfer interactions between inorganic anions, particularly the iodide ion and pyridinium, and substituted pyridinium cations, to determine the sensitivity of their charge–transfer absorption to the solvent environment, as well as the potential role of structures of this type in enzymatic oxidation-reduction processes [15]. As the charge–transfer complexes are a simpler, cheaper, and more efficient tool of analysis than the other methods mentioned in the literature, charge–transfer interactions are an important subject employed in the determination of medicines in pharmaceutical and pure forms [16].

Many reports stated the interactions, in solution, between flavin mononucleotide, flavin adenine dinucleotide, or riboflavin and a variety of donors, including hydrocarbons [17], indoles [18], NADH [19], NADPH [19], purines and pyrimidines, as well as other compounds with no obvious donor properties. There is little doubt that complete electron transfer happens in several of these systems to generate the flavin semiquinone [20]. The new broad absorption band reported for mixes of the reduced form of flavin mononucleotide (FMNH2) and (FMN) was attributed to the creation of charge–transfer complexes [21]. 2-methyl-1,4-naphthoquinone, also known as vitamin K3, used as a synthetic substitute for K1, o-quinone adrenochrome, and many other biologically important quinones have substantial electron donor complexing capacity [22].

Tryptophan appears to be unique among amino acids in its capacity to generate charge transfer complexes due to the strong donor characteristics of the indole ring. However, another study has shown that a pyridinium model compound of NAD+ may form complexes with tyrosine and phenylalanine [23]. Spectral evidence was also found to produce charge–transfer complexes between NAD+ and model pyridinium compounds with chymotrypsinogen, a tryptophan-rich protein [24].

Molecular docking (MD) is a computer method for efficiently predicting the non-covalent binding of macromolecules (receptors) and small molecules (acceptors) based on their unbound structures, structures generated through MD simulations, homology modeling, and other methods. The prediction of small molecule binding to proteins is of particular practical significance since it is used to screen virtual libraries of drug-like compounds for leads for further drug development. As a result, MD has become an important method in drug development.

Here, we used the Autodock Vina program to investigate the interactions between the ligand (SRX and synthesized CT complexes) and receptors (serotonin, dopamine, and TrkB kinase receptors). In the 1970s and 1980s periods, selective serotonin reuptake inhibitors (SSRIs) were developed, which are as effective antidepressants as tricyclics but do not have as many side effects as other antidepressant drugs. Binding energy, along with hydrophobic properties, ionizability, aromatic, and hydrogen bond surfaces, were also investigated. The molecular dynamic simulation was achieved at 300 K for 100 ns. The dynamic properties of the complexes were compared in many characterizations such as residue flexibility, structural solidity, solvent-accessible surface area, and other measurements. DFT using the B-3LYP/6-311G++ (basis set) level of theory was employed to obtain an optimized geometry of the CT complex- [(SRX)(PA}], [(SRX)(DNB)], [(SRX)(p-NBA)], [(SRX)(DCQ)], [(SRX)(DBQ)], and [(SRX)(TCNQ)] with minimal energy. Different parameters of the complexes were obtained and compared.

## 2. Materials and Methods

### 2.1. Synthesis of [(SRX)(π-Acceptor)] Charge–Transfer Complexes

**Figure 1:**
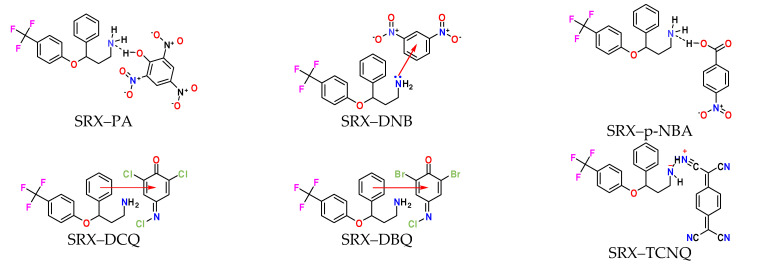
Speculated molecular structures of (1:1) charge-transfer complexes [(SRX)(π-acceptor)].

The charge–transfer complexes [(SRX)(π-acceptor)] where π-acceptor are PA, DNB, *p*-NBA, DCQ, DBQ, and TCNQ (Figure 1) were synthesized as 1:1 by the reaction of SRX donor in a solution (25 mL) of each acceptor [25].

At room temperature, the mixtures were agitated for about an hour in each case. The precipitate was filtered and washed with the smallest amount of dichloromethane possible before being dried under vacuum over anhydrous CaCl_2_.

### 2.2. Instruments and Measurements

With safeguards (platinum pans, nitrogen gas flow, and 30 °C min^−1^ heating rate), thermogravimetric analysis (TGA/DTG) was examined using Shimadzu TGA-50H equipment. A Perkin–Elmer Precisely Lambda 25 UV/Vis Spectrometer was used to scan the electronic absorption spectra of the synthesized charge–transfer complexes in the 200–800 nm region. A Bruker 600 MHz spectrometer was used to measure ^1^H-NMR spectra in DMSO solvent.

### 2.3. Molecular Docking

The structures of the SRX drug and CT complexes were handled in PDBQT format via OpenBabelIGUI software (version 2.4.1) [26]. Then, the PyRx-Python prescription 0.8 and MMFF94 force field were used to minimize the energy of the structure for 500 steps [27]. The RCSB Protein Data Bank [28] was used to get the 3D crystal structures of the three receptors. The receptors were arranged using the BIOVIA Discovery Studio Visualizer (v19.1.0.18287). Kollman charges were also measured with the help of the AutoDock Tool [29]. The Geistenger method was used to allocate partial charges. The docking calculations were performed with Autodock Vina [30]. The DS (Discovery Studio) Visualizer was used to examine the docked poses that resulted.

### 2.4. Molecular Dynamics (MD) Simulation

**Figure 2:**
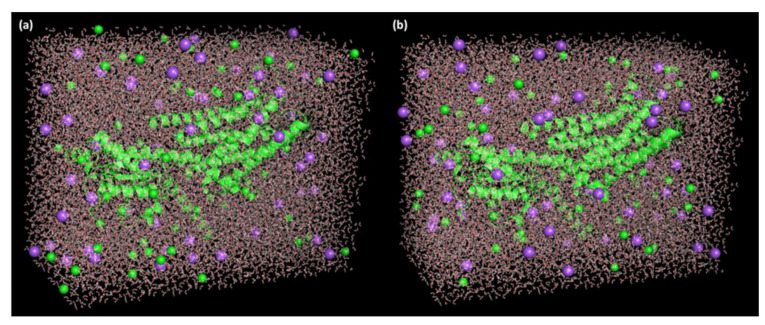
Receptor–ligand complex (**a**) SRXD and (**b**) CTcD in triclinic box solvated with water molecules and neutralized with 59 Na^+^ and 64 Cl^−^ ions (0.15 M salt).

The optimal receptor–ligand complex pose for SRX and [(SRX)(TCNQ)] with a maximum docking score was acquired through the molecular docking investigation. The GROMACS package version (2019.2) was used to accomplish MD simulation analysis via GROMOS96 43a1 force field. The parameter files and topologies were created with the most recent CGenFF through CHARMM-GUI [31,32]. The SPC water models that prolonged 10 Å from the receptor were utilized to explain receptor–ligand structures [33]. To neutralize the systems, 59 Na^+^ and 64 Cl^−^ ions (0.15 M salt) were injected to simulate physiological salt concentrations (Figure 2).

Both systems were exposed to periodic boundary conditions at a continuous temperature (300 K) and pressure (1.0 bar) for 100 ns simulation time with a Leap-frog MD integrator [34]. To minimize poor contact inside the system, energy reduction with 5000 steps was performed [35]. The gmx hbond device was used to investigate hydrogen bonding. The gyration radius was measured using gmx gyrate tool, while the solvent-accessible surface area was calculated by gmx sasa. The root mean square deviation (RMSD) of the protein was designed using the gmx rms tools. The GROMACS analytic tools [36] were used to accomplish trajectory analysis. Grace Software was used to compute the plots, while PyMol/VMD was utilized to visualize them [37].

### 2.5. Computational Structural Analysis

DFT (Density functional theory) computational study was used for structural analysis of CT complexes and optimized geometry with atomic coordinates, strain-free lattice constants, and ground state minimum energy structure are obtained. Gaussian 09RevD.01 program [38] was used for this study. Gradient corrected correlation was applied with Pople’s basic set B3LYP/6-311G++ [39]. For visualization of obtained DFT results, ChemCraft 1.5 software [40] was used.

## 3. Results and Discussion

### 3.1. Preapprehension

The attachment of the receptor to drugs does not affect the efficiency of its work, in fact, it improves it. However, it should be noted that different drugs have varying efficacy when they are connected with the receptor’s site [41,42,43,44,45]. Several reports showed differences in the efficacy of two drugs targeting the same receptor because the activation of the receptor is dependent on the rate of drug interaction with the receptor [43,44].

This drew pharmacologists’ attention to the importance of knowing the relationship between drug chemical composition and physiological action. These findings may aid our understanding of the molecular nature of drug–receptor interactions [43,44].

In many cases, the drug’s binding to the receptor seems to have low energy, certainly lower than that involved in conventional covalent bonding [45]. Ionic association, particularly hydrogen bonding, and other weaker forces such as charge–transfer forces, or a combination of many of these forces, can produce what is termed “receptor-drug complexing”. The capacity of drugs and related compounds to form charge–transfer complexes with well-defined electron acceptors or electron donors, primarily in non-aqueous circumstances, is used as a primary criterion for determining whether charge–transfer forces are manipulated in any way [46,47,48,49].

The λ_max_ of UV–Vis spectra of the synthesized charge–transfer complexes were found to be at 340 and 436 nm for (SRX)(PA), 351 nm for (SRX)(DNB), 353 nm for (SRX)(*p*BBA), 528 nm for (SRX)(DCQ), 540 nm for (SRX)(DBQ), and lastly 745 and 833 nm for (SRX)(TCNQ). According to photometric titration measurements, the produced charge–transfer complexes between SRX and corresponding π-acceptors had a 1:1 molar ratio. The dative structure D+–A of charge–transfer complexes in polar solvents were shown to be destabilized by the dissociation of charge–transfer complexes into D+ and A [50,51,52,53].

In pharmacokinetics, examining the physical and chemical properties of pharmacological substances in solution, as well as their mechanism of action, is critical. Spectroscopic and thermodynamic approaches are used to assess the binding strength of pharmaceutical compounds to other substances in living systems [41]. In biological and bioelectrochemical energy transfer processes, electron acceptor complexes (EDA) are a common occurrence [42]. The development of highly colored charge–transfer complexes is often related to molecular interactions between electron donors and acceptors, which absorb light in the visible area [48].

Electron acceptor complexes with ionic bands are the most prevalent. Ionic interactions and structural recognition are two crucial mechanisms in biological systems. For example, drug action, enzyme activation, and ion transport across lipophilic membranes are all intricate [45]. Ionic interactions are the fundamental outputs of selectivity, rate control, and reversibility in many biological systems [46].

The most commonly used procedures for assessing various drugs and sophisticated charge transfer investigations include UV direct spectrophotometry [47], colorimetry [48], and HPLC [49]. EDA compounds, as previously reported, have good nonlinear optical properties and electrical conductivity [54].

The six charge–transfer complexes were expected to have particle sizes of 50 nm for (SRX)(PA), 25 nm for (SRX)(DNB), 5 nm for (SRX)(*p*NBA), 10 nm for (SRX)(DCQ), 20 nm for (SRX)(DBQ), and 5 nm for (SRX)(DBQ) (TCNQ). These findings were based on TEM scans, which showed that the particles of the manufactured charge–transfer were nanoscale in size.

**Figure 3:**
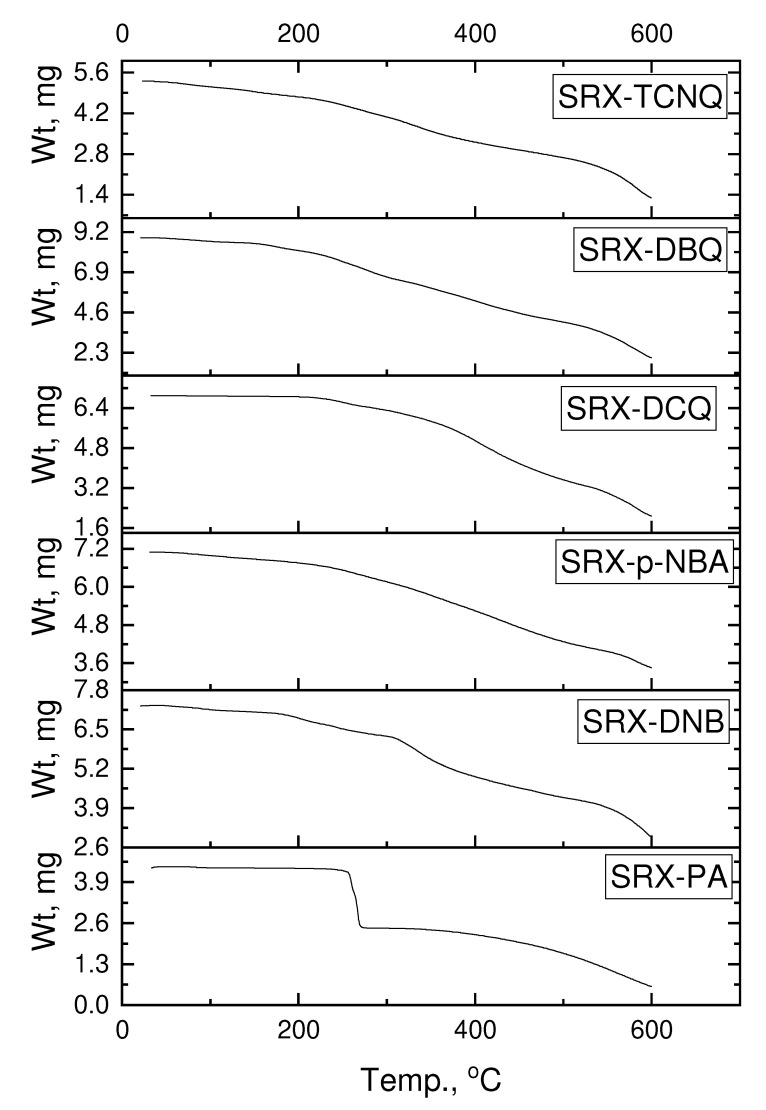
TGA curves of (1:1) charge-transfer complexes [(SRX)(π-acceptor)].

The simultaneous thermal stability on the TG/DTG curves of all charge–transfer complexes at a heating rate of 10 °C/min in a static nitrogen atmosphere are shown in Figure 3. The overall mass loss from the TGA curves was 78.17% for SRX–PA, 58.38% for SRX–DNB, 50.45% for SRX-p-NBA, 69.40% for SRX–DCQ, 77.58% for SRX–DBQ, and 75.69% for the SRX–TCNQ complexes. The complexes had mass losses of one to three maxima peaks. The thermal analysis of the curves of the [(SRX)(π-acceptor)] CT complexes clearly shows that the maximum DTG peaks are located at 415, 230, 357, 383, 343, and 370 °C, respectively.

**Figure 4:**
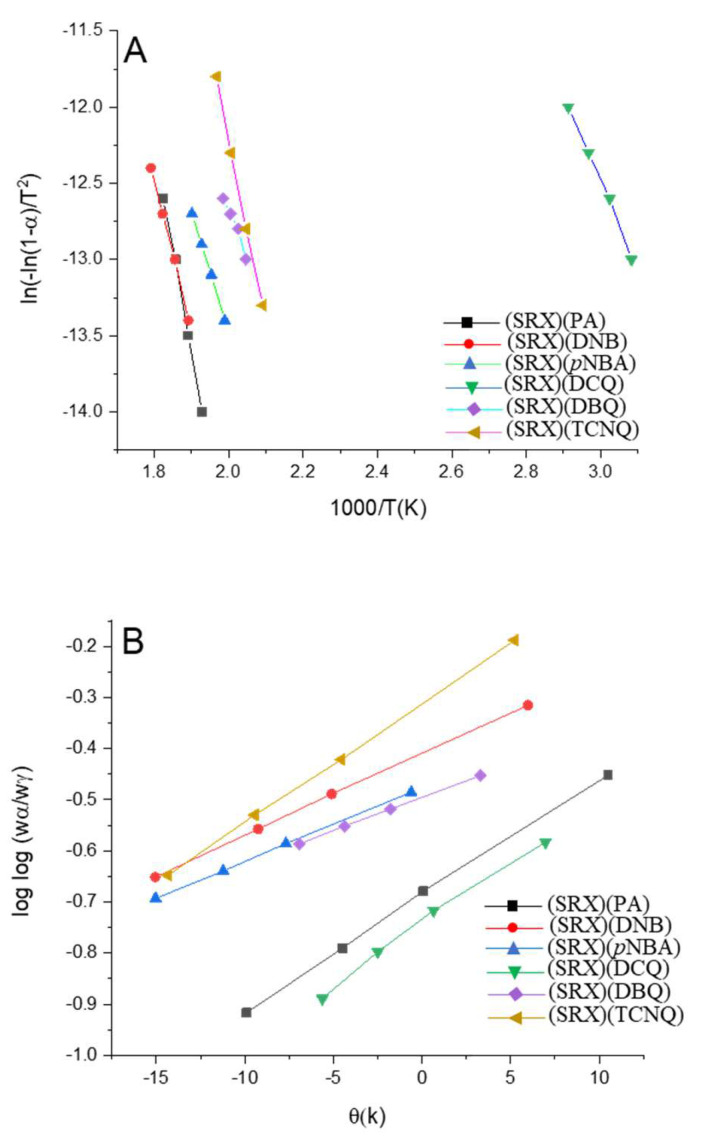
Kinetic curves of (1:1) charge-transfer complexes [(SRX)(π-acceptor)] using (**A**) Coats-Readfern and (**B**) Horowitez-Metzegar methods.

The Coats-Readfern and Horowitez-Metzegar methods [55,56] were used to collect the kinetic thermodynamic data of the maximal DTG peak decomposition steps of all charge–transfer complexes. The kinetic parameters, *E*, *A*, Δ*S*, Δ*H*, Δ*G*, and *r* were calculated, and the data are listed in Table 1 and displayed in Figure 4.

The activation energies of the [(SRX)(π–acceptor)] CT complexes in the case of the maximum DTG peak decomposition step were as follows:

(SRX)(TCNQ) > (SRX)(PA) > (SRX)(DNB) > (SRX)(*p*NBA) > (SRX)(DBQ) > (SRX)(DCQ).

Among the six π–acceptors, it was found that the SRX–TCNQ and SRX–PA complexes had greater activation energies than the other charge–transfer complexes. This is owing to the presence of cyano and nitro groups in the TCNQ and PA acceptors [57].

### 3.2. UV–Vis Spectra and Photometric Titration

**Figure 5:**
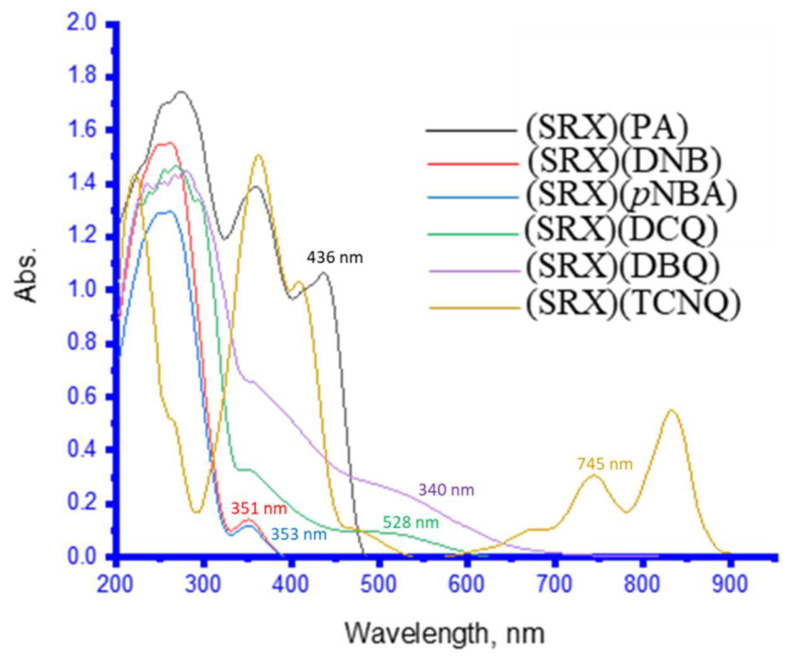
UV–Vis spectra curves of the SRX with the six π–acceptors complex [4].

**Figure 6:**
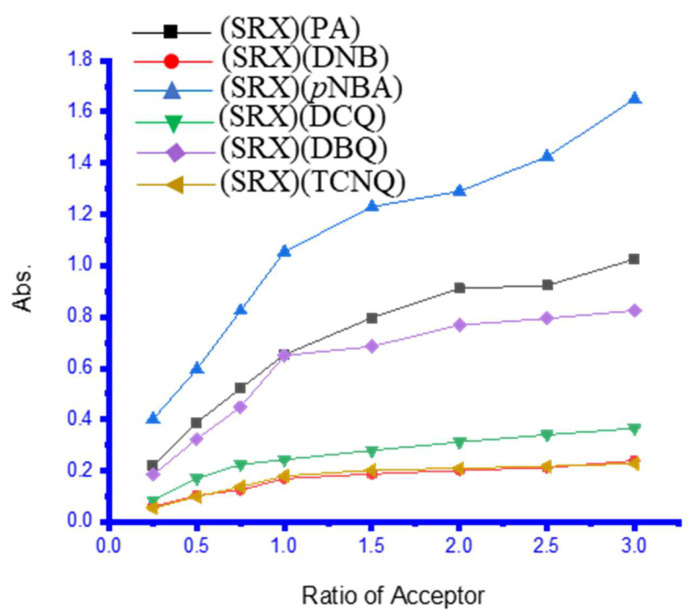
Photometric titration curves of the SRX with the six π–acceptors complex [4].

The UV-Vis spectra of the six charge–transfer complexes in methanol solvent were investigated in the 200–900 nm range (Figure 5) [4]. These charge–transfer complexes are formed by combining 1.00 mL of 0.5 mM from the SRX drug donor with different volumes of the six π-electron acceptors to reach a final concentration of 0.5 mM. With methanol as the solvent, each charge–transfer system had a total volume of 5 mL. Absorption bands for [(SRX)(PA), [(SRX)(DNB)], [(SRX)(p-NBA)], [(SRX)(DCQ)], [(SRX)(DBQ)], and [(SRX)(TCNQ)] donor–acceptor interaction systems appeared at λmax of 436 nm, 351 nm, 353 nm, 528 nm, 540 nm, and 745 nm, respectively. At 25 °C, photometric titrations were performed with the SRX medication as an electron donor and the six π–electron acceptors. The molar ratio of the produced charge–transfer complexes between SRX and the corresponding π–electron was 1:1. The photometric titration curves for the maximal charge–transfer absorption bands (λmax) are shown in Figure 6 [4].

The photometric titration findings were obtained by graphing the absorbance (Y-axis) against the ratio of indicated acceptors (X-axis) using established procedures [4].

The molar ratio of the produced charge–transfer complexes between SRX medication and identified–acceptors is 1:1 (Figure 6).

### 3.3. ^1^H-NMR Spectra

**Figure 7:**
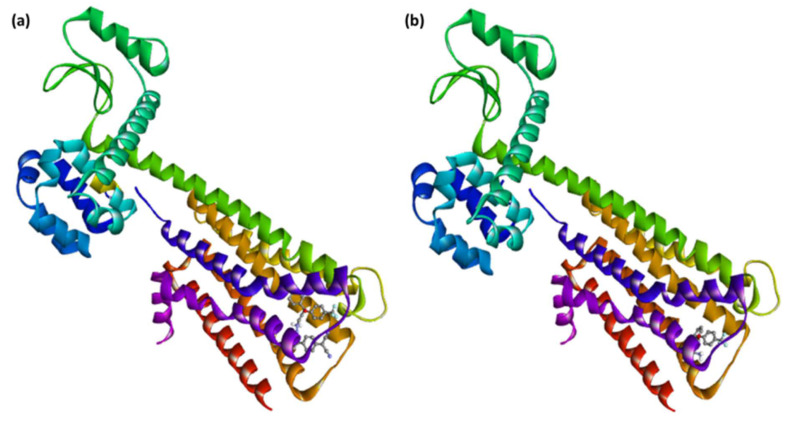
Best docking pose showing a helical model of dopamine docked with (**a**) [(SRX)(TCNQ)] and (**b**) [SRX].

The ^1^H-NMR spectra of all six π-acceptors complexes are investigated (Figure 7); while the 1H-NMR spectra of SRX only were cited previously [58]. The NH_2_ protons of the SRX amino group are downfield displaced by 6.87–6.98 ppm as a result of the involvement of one pair of electrons on the amino group towards the six electron π-acceptors. The peaks of other aromatic and methylene protons are similarly pushed downfield to higher ppm values, indicating the formation of six charge–transfer complexes (Supplementary Material Figure S1).

### 3.4. Molecular Docking Studies

To find the optimal docking pose, the six CT complexes were docked against three protein receptors: serotonin, dopamine, and TrkB kinase. For comparison, the SRX drug (donor moiety) was employed as a control. The potential binding energy of CT complexes was higher than that of SRX in all receptors, according to the molecular docking of these six complexes (Table 2).

Of the six CT complexes studied, [(SRX)(TCNQ)] exhibited the highest docking energy values. [(SRX)(TCNQ)] had predicted binding energies of −9.3, −9.9, and −8.2 kcal/mol with serotonin, dopamine, and TrkB kinase receptors, respectively. The binding energy of [(SRX)(TCNQ)]-dopamine (CTcD) is higher than that of serotonin and the TrkB kinase receptors, indicating a stronger link. The optimal docking pose of (CTcD) is shown in Figure 5, and the docking data are listed in Table 3.

**Figure 8:**
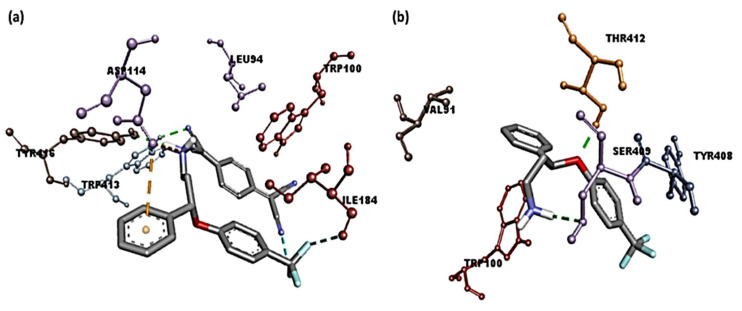
Three-dimesnional representation of interactions for dopamine docked with (**a**) [(SRX)(TCNQ)] and (**b**) [SRX].

The [(SRX)(TCNQ)]-dopamine (CTcD) shows that the amino acid residues, including Tyr416 and Trp413, formed hydrogen bond interactions (Figure 8a). There are other interactions between Leu94, Trp100 (π-Alkyl), Phe189 (π-Sigma), Asp114 (π-Anion), and Ile184 (halogen-fluorine) [59]. The theoretical binding energies of SRX with the serotonin, dopamine and TrkB kinase receptors were −7.3, −7.4, and −6.0 kcal/mol, respectively, after molecular docking. The [SRX]-dopamine (SRXD) receptor had a stronger connection than the serotonin and TrkB kinase receptors due to its greater binding energy value.

The interaction between SRX and dopamine is illustrated in Figure 8b. The amino acid residues, including Ser409 and Thr412, formed hydrogen bond connections between SRX and dopamine. There were also interactions between Tpr100, Val91 (π-alkyl), and Tyr416 (π-sigma). These data indicate that the [(SRX)(TCNQ)] complex binds to the three protein receptors more efficiently than the reactant donor (SRX) alone and that the CTcD has the highest binding energy value. TNCQ is a powerful electron acceptor that forms charge transferring chains due to the existence of its four cyano groups and π-conjugation bonds. This facilities the increase in interactions (such as H-bond, π-Alkyl, π-Sigma, π-Anion, along with SRX) with receptors.

**Figure 9:**
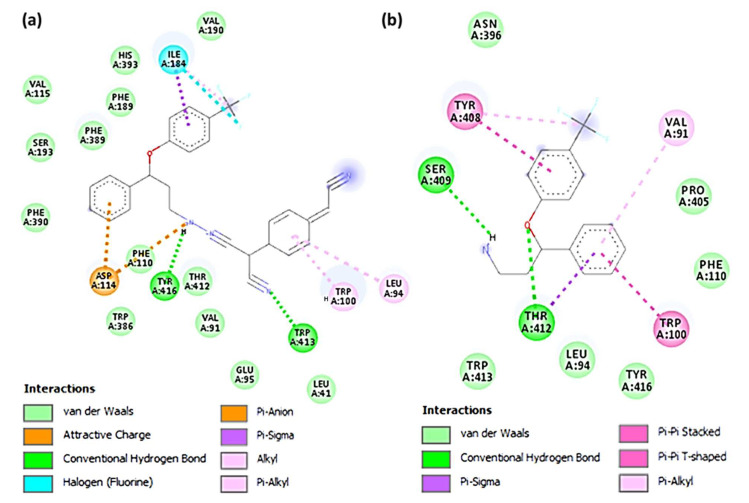
Two-dimensional representation of interactions of dopamine docked with (**a**) CT complex and (**b**) SRX.

**Figure 10:**
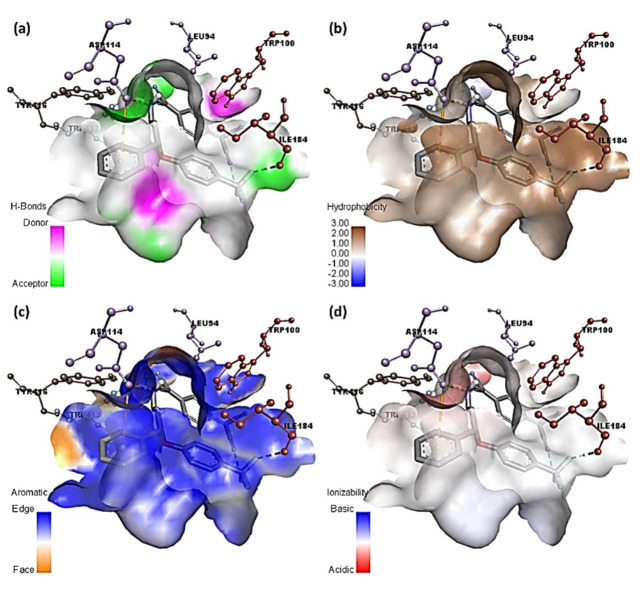
Representation of (**a**) hydrogen binding surface, (**b**) hydrophobic surface, (**c**) aromatic surface, and (**d**) ionizability surface; between dopamine and CT complex.

Given the growing evidence that DA transmission assists antidepressant therapeutic goals [60], this augmentation of transmission could have clinical implications. This is because the majority of modern antidepressants do not boost dopamine neurotransmission [60]. One reason for DA’s significance is that it regulates motivation, concentration, and pleasure [60]. Figure 9 shows two-dimensional depictions of ligand–receptor interactions. Figure 10 and Figure S2 show the hydrophobic, ionizability, aromatic, and hydrogen bond surfaces at the interaction location of [(SRX)(TCNQ)] and dopamine, respectively.

### 3.5. Molecular Dynamics Simulation

**Figure 11:**
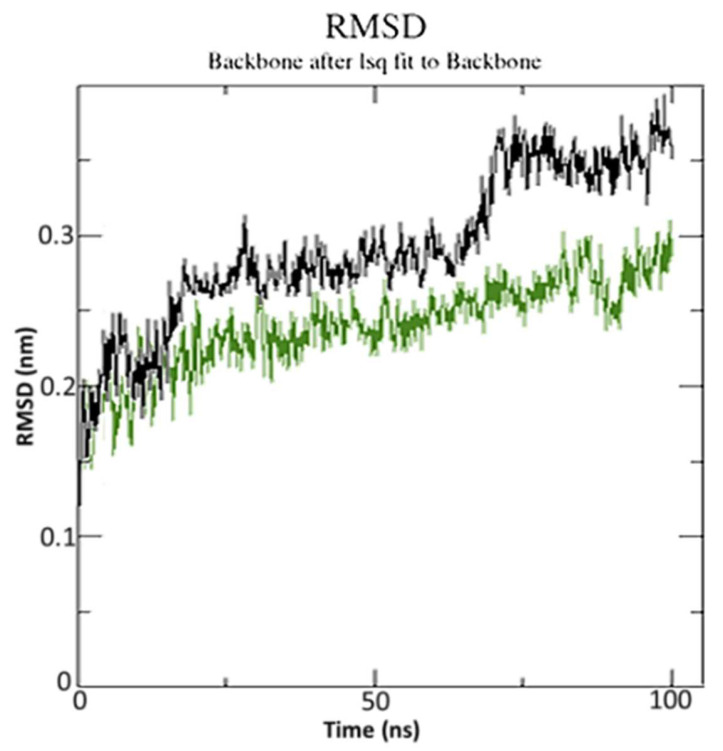
The root mean square deviation (RMSD) of solvated receptor backbone and ligand complex during 100 ns MD simulation [SRXD complex (black) and CTcD complex (green)].

For the 100 ns simulation run, the best-docking position for SRXD and CTcD with the highest docking score was used. The RMSD of molecular dynamics data was calculated to investigate structural stability. After 45 ns and 60 ns, respectively, SRXD and CTcD established constant conformation with an appropriate RMSD value of 2.85 and 3.56, respectively (Figure 11).

**Figure 12:**
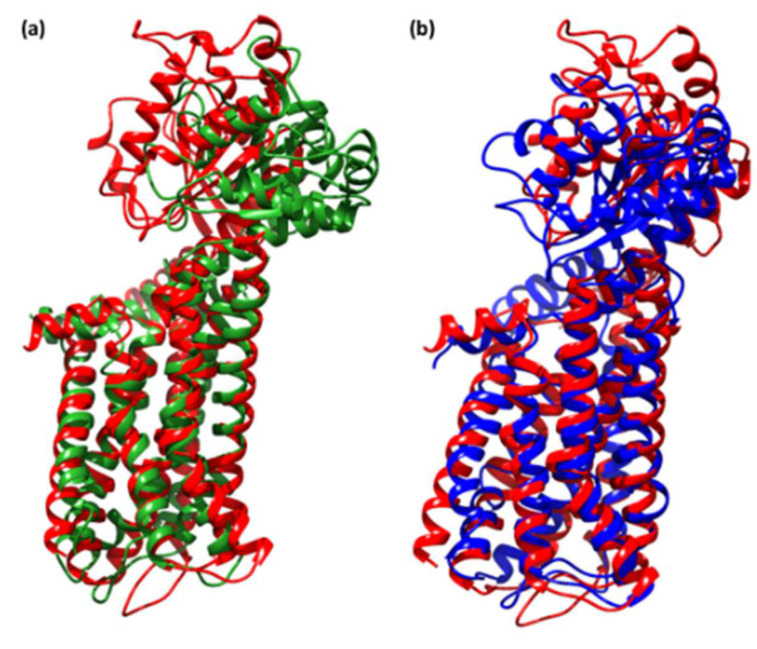
Superimposed structure after simulation of unbound dopamine receptor (red) and (**a**) CTcD (green), and (**b**) SRXD (blue).

As indicated previously, <3.0 Å is the most acceptable RMSD value range, which indicates better system stability [61]. This finding shows that the CTcD develops a more stable combination. The findings revealed that ligand-receptor interactions bring protein chains closer and reduce the gap between them, as shown in Figure 12 [62].

**Figure 13:**
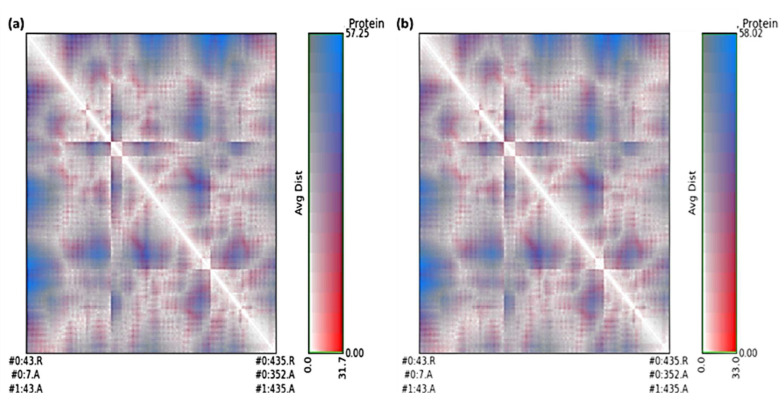
RR distance map between unbound dopamine receptor and after simulation for SRXD (**a**), and CtcD (**b**).

The average distance and standard deviation for all amino acid pairs between two conformations were calculated using RR distance maps [63]. In Figure 13, the patterns of spatial interactions are depicted using the RR distance maps [64].

**Figure 14:**
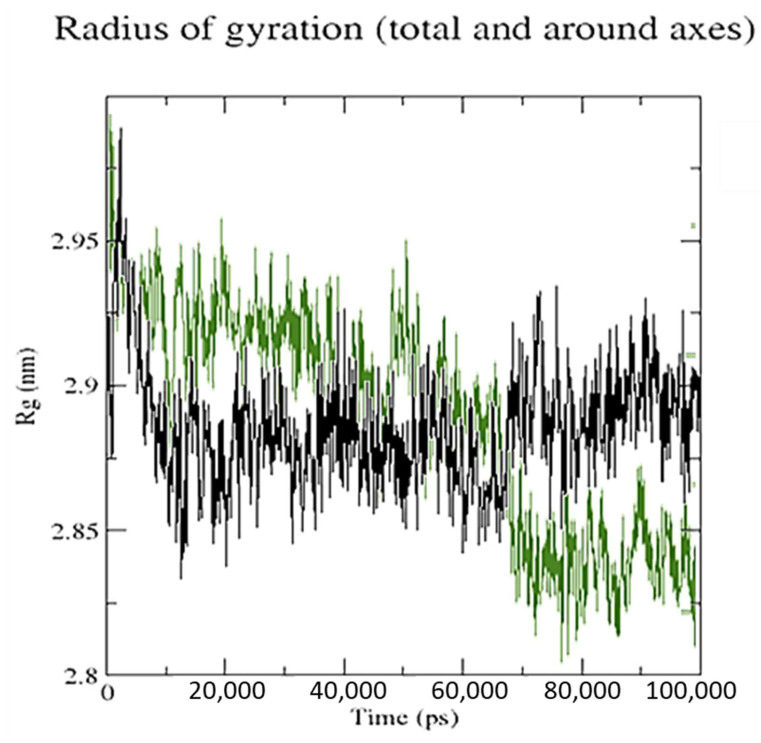
The radius of gyration (Rg) for SRXD complex (black) and CTcD complex (green) during 100 ns simulation time.

On the map, the white oblique represents the zero distance between two amino acid residues, whereas the red and blue elements depict residue pairs with the biggest distance deviations between the two forms. The average radius of gyration (Rg) value of 28.75 and 28.52 Å was observed for SRXD and CTcD, respectively. Along the simulation time, Rg decreased, indicating that the structures became more compact (Figure 14).

**Figure 15:**
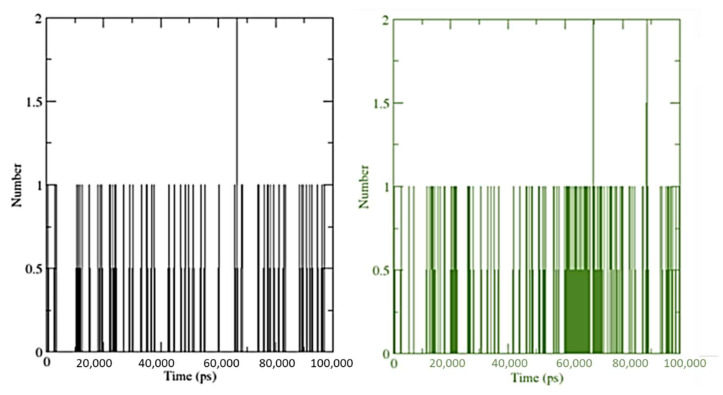
Number of average hydrogen bonding interactions between (**Left**) SRXD complex and (**Right**) CTcD complex during 100 ns simulation time.

The number of hydrogen bond interactions between ligand and receptor combinations (SRXD and CTcD) were displayed against time using a grid search on a 15 × 20 × 27 grid with a rcut = 0.35 value (Figure 15).

The hydrogen bonds between SRX and dopamine were at 33 and 1356 atoms, respectively. While they were between 56 and 5109 atoms for the CT complex and dopamine. However, there were 709 donors for both (SRXD and CTcD), 1356 acceptors for SRXD, and 1426 acceptors for CTcD. For SRXD and CTcD, the average number of hydrogen bonds per time was found to be 0.065 and 0.144 out of 480,702 possible.

**Figure 16:**
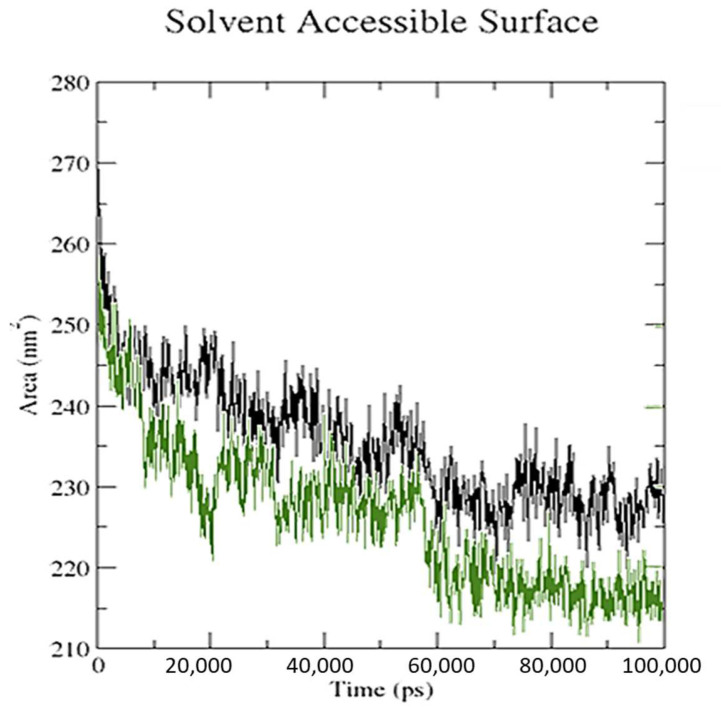
Solvent accessible surface area analysis for the SRXD complex (black) and the CTcD complex (green) during 100 ns simulation time.

Overall, these findings suggest that the receptor–protein interaction increased the number of hydrogen bonds by a significant amount in CTcD. As the ligand attached to the receptor, the values of the solvent-accessible surface area (SASA) changed (Figure 16). When the receptor interacts with the ligand, the SASA is lowered, indicating a change in protein structure and a smaller pocket size with increased hydrophobicity.

### 3.6. Theoretical Structural Analysis

**Figure 17:**
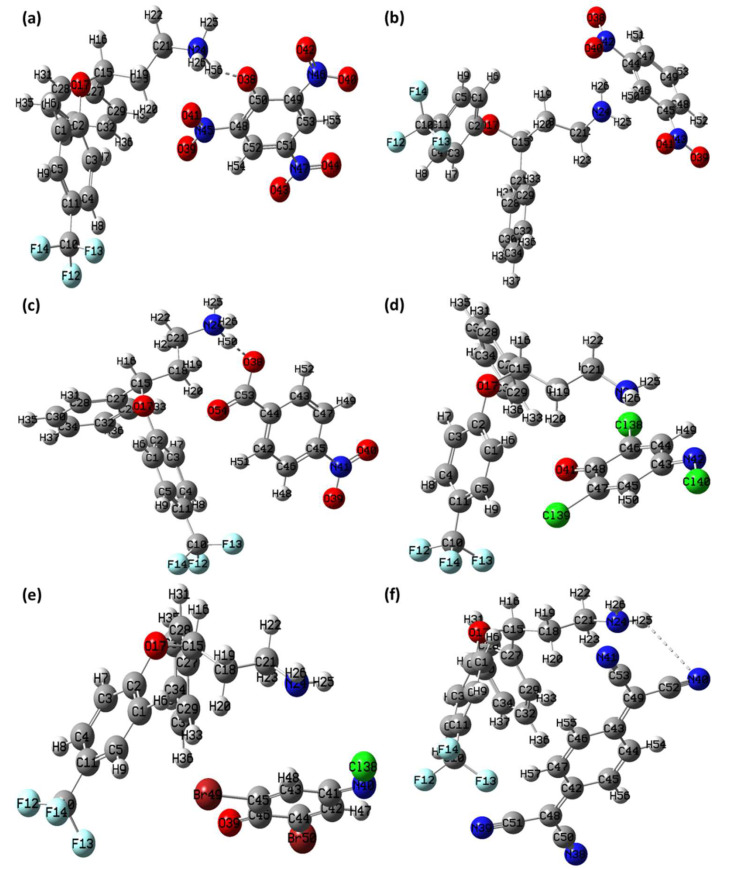
Optimized structure of (**a**) [(SRX)(PA}], (**b**) [(SRX)(DNB), (**c**) [(SRX)(p-NBA)], (**d**) [(SRX)(DCQ)], (**e**) [(SRX)(DBQ)], and (**f**) [(SRX)(TCNQ)] with Mulliken atom numbering scheme.

**Figure 18:**
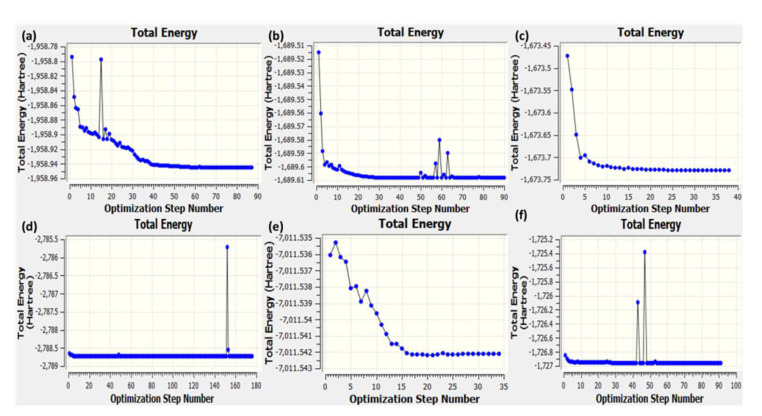
Optimization step graph for (**a**) [(SRX)(PA}], (**b**) [(SRX)(DNB), (**c**) [(SRX)(p-NBA)], (**d**) [(SRX)(DCQ)], (**e**) [(SRX)(DBQ)], and (**f**) [(SRX)(TCNQ)].

Density functional theory (DFT) using B-3LYP/6-311G++ (basis set) level of theory and optimized geometry of the CT complexes- [(SRX)(PA}], [(SRX)(DNB), [(SRX)(p-NBA)], [(SRX)(DCQ)], [(SRX)(DBQ)], and [(SRX)(TCNQ)] with atomic coordinates, strain-free lattice constants and ground state minimum energy structure are obtained. The optimized structures of all the CT complexes with the Mulliken numbering scheme are shown in Figure 17. The minimum SCF energy of obtained for [(SRX)(PA}], [(SRX)(DNB), [(SRX)(p-NBA)], [(SRX)(DCQ)], [(SRX)(DBQ)], and [(SRX)(TCNQ)] is −1958.944644 to, −1689.608194, −1673.728419, −2788.736562, −7011.542112, and −1726.964350 a.u in 87, 90, 38, 176, 34, and 91 steps, respectively (Figure 18). Based on the optimized structure, some molecular parameters (SCF minimum energies, dipole moments, and Electronic spatial extent) were calculated in the gas phase (Table 4). The HOMO–LUMO gap (∆E) for [(SRX)(PA}], [(SRX)(DNB), [(SRX)(p-NBA)], [(SRX)(DCQ)], [(SRX)(DBQ)], and [(SRX)(TCNQ)] was calculated as 2.78, 3.44, 3.31, 2.29, 2.43, and 1.89 eV, respectively. The overall order of the chemical reactivity of the CT complexes on the bases of ∆E is as follows- [(SRX)(TCNQ)] > [(SRX)(DCQ)] > [(SRX)(DBQ)] > [(SRX)(PA}] > [(SRX)(p-NBA)] > [(SRX)(DNB)].

## 4. Conclusions

The charge transfer complexes between the seproxetine as a donor and picric acid, dinitrobenzene, p-nitrobenzoic acid, 2,6-dichloroquinone-4-chloroimide, 2,6-dibromoquinone-4-chloroimide, and 7,7′,8,8′-tetracyanoquinodi methane as π-electron acceptors were characterized and studied for interaction with three receptors (serotonin, dopamine, and TrkB kinase receptor). The spectrophotometric analysis confirmed that the charge–transfer interactions between the electrons of the donor and acceptors were 1:1 (SRX: *π*–acceptor). Molecular docking revealed that the CT complex [(SRX)(TCNQ)] interacted with all three receptors more efficiently than the reactant donor (SRX); among all, [(SRX)(TCNQ)]-dopamine (CTcD) had the highest binding energy value. Using AutoDock Vina, the molecular dynamics simulation of the 100 ns run revealed that both the SRX-dopamine and CTcD complexes had a stable conformation; however, the CTcD complex was more stable. DFT calculations provided the optimized geometries of the CT complexes. In the context of mounting evidence for the role of DA transmission, such transmission enhancement might be of potential research and clinical benefit.

## Figures and Tables

**Table 1:** Kinetic thermodynamic parameters for the six charge–transfer complexes based on Coats–Redfern (CR) and Horowitz–Metzger (HM) methods.

Complex	Method	Parameter					r
		*E* (kJol^−1^)	*A* (s^−1^)	Δ*S* (J mol^−1^ K^−1^)	Δ*H* (kJ mol^−1^)	Δ*G* (kJ mol^−1)^	
(SRX)(PA)	CR	11.5 × 104	4.00 × 108	−8.52 × 101	1.12 × 105	1.54 × 105	0.9990
	HM	11.2 × 104	5.60 × 109	−6.32 × 101	1.12 × 105	1.50 × 105	0.9989
(SRX)(DNB)	CR	7.80 × 104	1.50 × 105	−1.55 × 102	7.25 × 104	1.47 × 105	0.9980
	HM	8.65 × 104	1.34 × 105	−1.30 × 102	8.12 × 104	1.44 × 105	0.9989
(SRX)(*p*NBA)	CR	6.38 × 104	1.32 × 104	−1.72 × 102	5.90 × 104	1.51 × 105	0.9995
	HM	7.23 × 104	1.22 × 104	−1.56 × 102	6.71 × 104	1.54 × 105	0.9985
(SRX)(DCQ)	CR	4.80 × 104	1.25 × 105	−1.45 × 102	4.43 × 104	9.40 × 104	0.9943
	HM	5.22 × 104	1.85 × 106	−1.32 × 102	4.68 × 104	9.22 × 104	0.9987
(SRX)(DBQ)	CR	5.77 × 104	5.12 × 103	−1.85 × 102	5.22 × 104	1.45 × 105	0.9890
	HM	6.35 × 104	2.75 × 104	−1.72 × 102	5.90 × 104	1.40 × 105	0.9994
(SRX)(TCNQ)	CR	11.1 × 104	6.22 × 108	−8.14 × 101	9.72 × 104	1.33 × 105	0.9984
	HM	11.8 × 104	5.50 × 109	−6.35 × 101	1.12 × 105	1.42 × 105	0.9996

**Table 2:** The docking score of six synthesized CT complexes docked with three receptors [serotonin (PDB ID: 6BQH), dopamine (PDB ID: 6CM4), and TrkB kinase (PDB ID: 4ASZ)].

Receptor	Binding Free Energy (kcal/mol)		
	6BQH	6CM4	4ASZ
SRX-PA	−7.8	−9.2	−8.4
SRX-DNB	−6.8	−8.3	−6.5
SRX-*p*NBA	−8.7	−7.8	−7.0
SRX-DCQ	−7.5	−9.5	−7.4
SRX-DBQ	−7.9	−8.1	−7.5
SRX-TCNQ	−9.4	−9.9	−8.2
SRX	−7.4	−7.3	−6.0

**Table 3:** The interactions of SRX-TCNQ and SRX with dopamine (6CM4).

Receptor	Binding Free Energy (kcal/mol)	Interactions	
		H-Bond	Others
SRX-TCNQ	−9.9	Tyr416 and Trp413	Leu94, Trp100 (π-Alkyl); Phe189 (π-Sigma); Asp114 (π-Anion); Ile184 (Halogen-Fluorine)
SRX	−7.3	Ser409 and Thr412	Tpr100, Val91 (π-Alkyl); Tyr416 (π-Sigma)

**Table 4:** Theoretical molecular parameters of the CT complexes obtained through DFT.

CT Complex	Minimum SCF Energy (a.u.)	Dipole Moment (Debye)	Electronic Spatial Extent (a.u.)	Δ*E* (eV)
[(SRX)(PA}]	−1958.944644	10.500053	33,762.8991	2.7845
[(SRX)(DNB)]	−1689.608194	9.644797	20,168.3034	3.4449
[(SRX)(p-NBA)]	−1673.728419	11.524028	26,521.9908	3.3189
[(SRX)(DCQ)]	−2788.736562	5.693616	19,344.1851	2.3924
[(SRX)(DBQ)]	−7011.542112	5.965700	18,542.9710	2.4310
[(SRX)(TCNQ)]	−1726.964350	5.607618	35,156.4199	1.8942

## Data Availability

All data supporting the reported results are available in the manuscript.

## Supplementary Materials

The following supporting information can be downloaded at: https://www.mdpi.com/article/10.3390/molecules27103290/s1, Figure S1: ^1^H-NMR spectrum of all six π–acceptors complexes; Figure S2: Representation of (a) hydrogen binding surface, (b) hydrophobic surface, (c) aromatic surface, and (d) ionizability surface; between dopamine and SRX.

## Sample Availability

Samples of the compounds are available from the corresponding author.
