# Supplementary material for: Increasing the Efficacy of Seproxetine as an Antidepressant Using Charge–Transfer Complexes
Source: Molecules. 2022 May 20;27(10):3290. doi: 10.3390/molecules27103290 (PMC9147639; doi:10.3390/molecules27103290)
Supplement: Supplementary file 1 [file molecules-27-03290-s001.zip › molecules-1723686-supplementary.pdf]

Supplementary Data

# Increasing the efficacy of seproxetine as an antidepressant using charge-transfer complexes

Walaa F. Alsanie[1,2], Abdulhakeem S. Alamri[1,2], Hussain Alyami[3], Majid Alhomrani[1,2], Sonam Shakya[4], Hamza Habeeballah[5], Heba A. Alkhatabi[6,7,8],Raed I. Felimban[5,9], Ahmed S. Alzahrani[2], Abdulhameed Abdullah Alhabeeb[10],Bassem M. Raafat[11], Moamen S. Refat[12,*], and Ahmed Gaber[2,13,*]

[1]Department of Clinical Laboratories Sciences, The faculty of Applied Medical Sciences, Taif University, P.O. Box 11099, Taif 21944, Saudi Arabia; w.alsanie@tu.edu.sa (W.F.A.); m.alhomrani@tu.edu.sa (M.A.); a.alamri@tu.edu.sa (A.S.A.); a.s.zahrani@tu.edu.sa (Ah.S.A.)
[2]Centre of Biomedical Sciences Research (CBSR), Deanship of Scientific Research, Taif University, P.O. Box 11099, Taif 21944, Saudi Arabia.
[3]College of Medicine, Taif University, Taif University, P.O. Box 11099, Taif 21944, Saudi Arabia; hmyami@tu.edu.sa (H.A.)
[4]Department of Chemistry, Faculty of Science, Aligarh Muslim University, Aligarh 202002, India; sonamshakya08@gmail.com (S.S.)
[5]Department of Medical Laboratory Technology, Faculty of Applied Medical Sciences in Rabigh, King Abdulaziz University, Jeddah 21589, Saudi Arabia; hhabeeballah@kau.edu.sa (H.H.)
[6]Department of Medical Laboratory Technology, Faculty of Applied Medical Sciences, King Abdulaziz University, Jeddah 21589, Saudi Arabia; halkhattabi@kau.edu.sa (H.A.A.); faraed@kau.edu.sa (R.I.F.)
[7]Center of Excellence in genomic Medicine Research (CEGMR), King Abdulaziz University, Jeddah 21589, Saudi Arabia.
[8]King Fahd Medical Research Centre, Hematology Research Unit, King Abdulaziz University, Jeddah 21589, Saudi Arabia.
[9]Center of Innovation in Personalized Medicine (CIPM), 3D Bioprinting Unit, King Abdulaziz University, Jeddah 21589, Saudi Arabia.
[10]National Centre for mental health promotion, P.O. Box 95459, Riyadh 11525, Saudi Arabia; aalhabeeb@ncmh.org.sa (A.A.A.)
[11]Department of Radiological Sciences, College of Applied Medical Sciences, Taif University, P.O. Box 11099, Taif 21944, Saudi Arabia; bassemraafat@tu.edu.sa (B.M.R.)
[12]Department of Chemistry, College of Science, Taif University, P.O. Box 11099, Taif 21944, Saudi Arabia; moamen@tu.edu.sa (M.S.R.)
[13]Department of Biology, College of Science, Taif University, P.O. Box 11099, Taif 21944, Saudi Arabia**;** a.gaber@tu.edu.sa (A.G.)

*****Correspondence: Correspondence: a.gaber@tu.edu.sa; moamen@tu.edu.sa

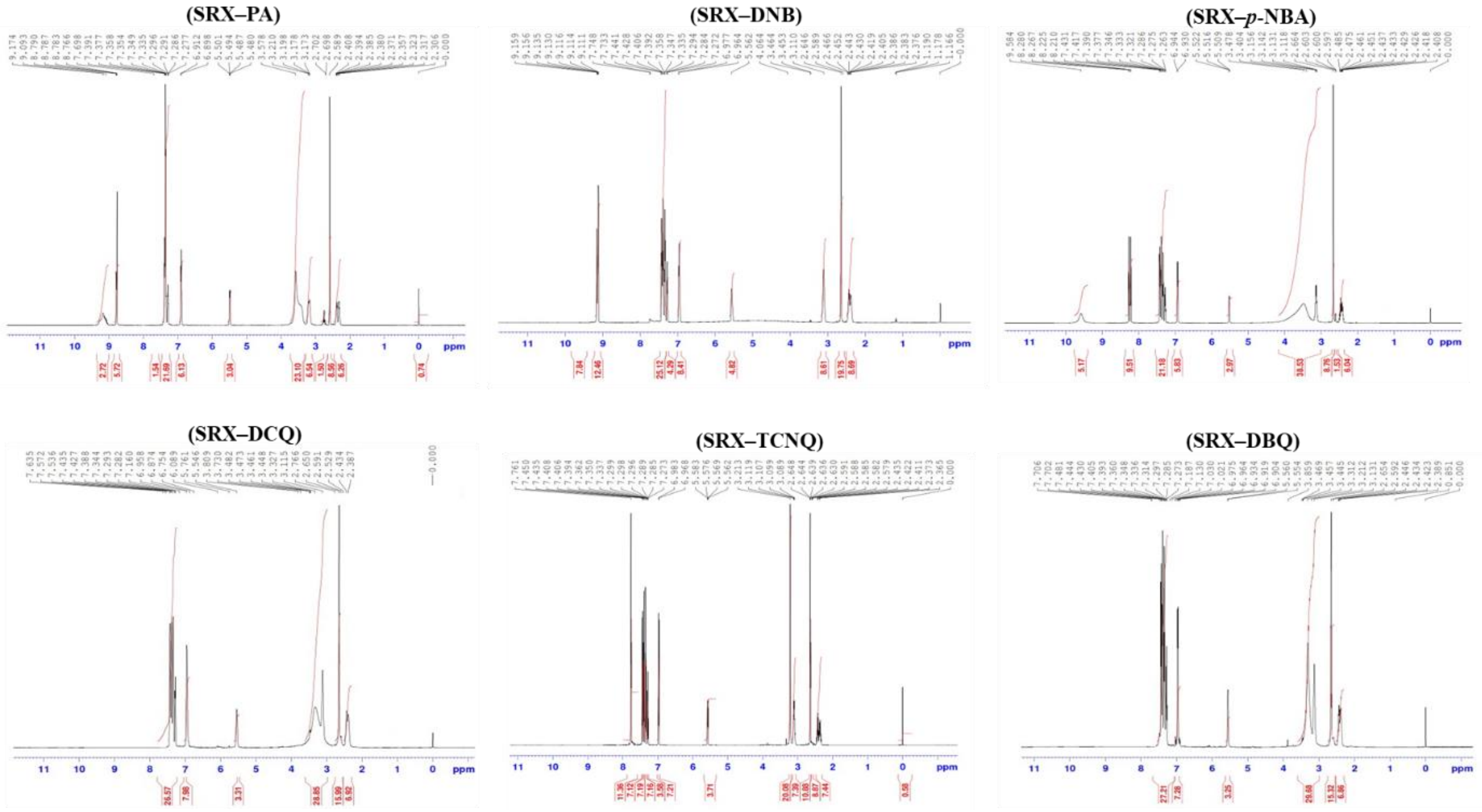


***F*igure S1.** $^{1}$H-NMR spectrum of all six π–acceptors complexes.

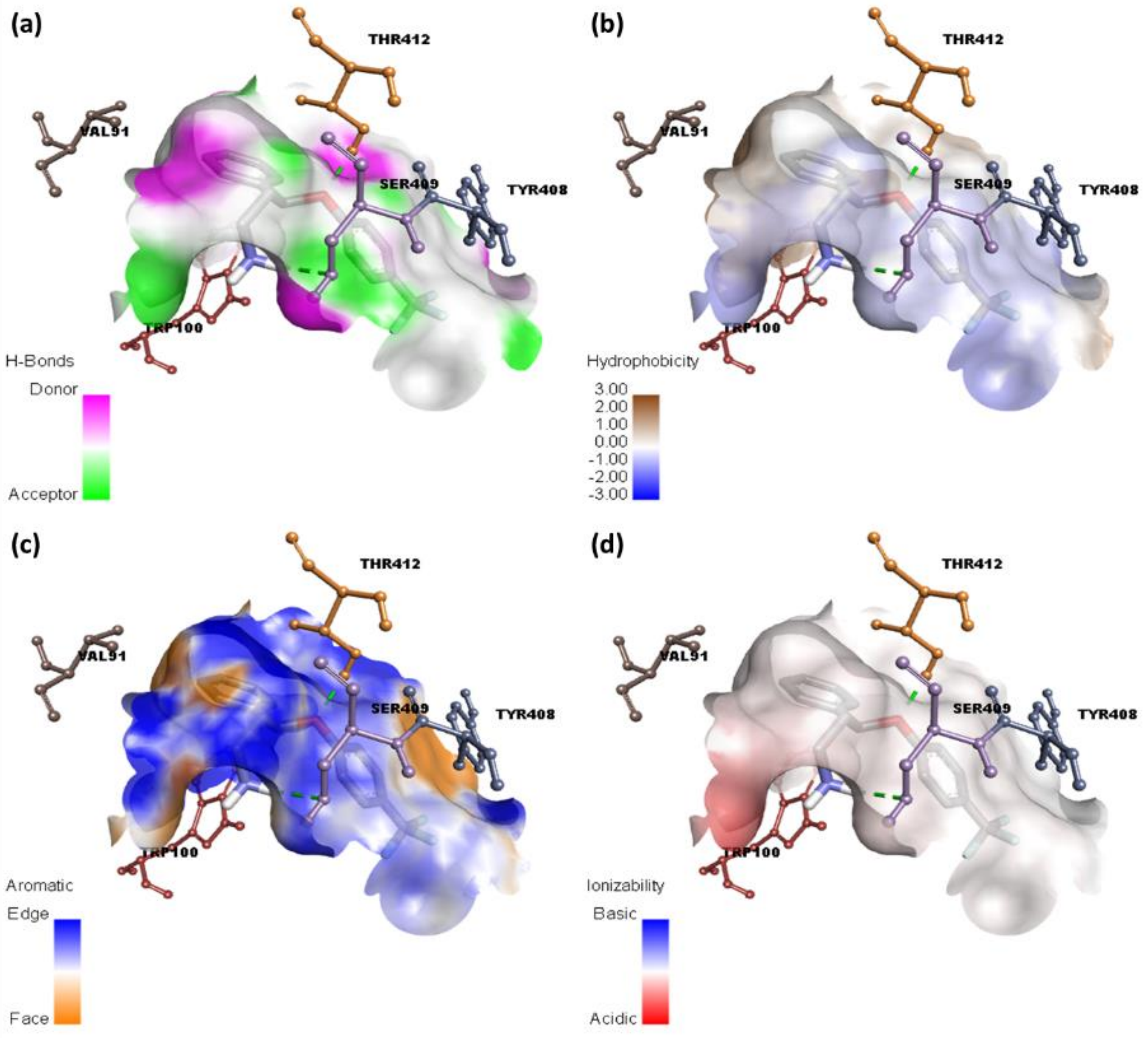


**Figure S2.** Representation of **(a)** hydrogen binding surface, **(b)** hydrophobic surface, **(c)** aromatic surface, and **(d)** ionizability surface; between dopamine and SRX.
